# Five steps for the deployment of artificial intelligence-driven healthcare delivery for remote and indigenous populations in Canada

**DOI:** 10.1177/20552076251334422

**Published:** 2025-04-13

**Authors:** Amal Khan, Sandro Galea, Ivar Mendez

**Affiliations:** 1 Department of Community Health and Epidemiology, University of Saskatchewan, Saskatoon, SK, Canada; 2 Remote Medicine and Robotics, Virtual Health Hub, Saskatoon, SK, Canada; 3 Margaret C. Ryan Dean of the School of Public Health, Washington University in St Louis, St Louis, MO, USA; 4 Department of Surgery, University of Saskatchewan, Saskatoon, SK, Canada

**Keywords:** AI and healthcare, AI-driven healthcare, digital health, healthcare delivery of diverse populations, indigenous populations, virtual care, rural communities

## Abstract

The integration of artificial intelligence (AI) into healthcare delivery offers transformative potential, especially for remote and underserved populations. In rural and remote regions like northern Saskatchewan, Canada, where Indigenous communities face elevated rates of chronic conditions such as diabetes and limited access to healthcare, AI-driven virtual care can bridge critical gaps. However, a universal approach falls short of addressing the unique needs of diverse populations. This communication outlines a five-step framework to guide AI-facilitated healthcare delivery tailored to community-specific demographics and clinical priorities. Steps include building comprehensive community profiles, assessing digital readiness, prioritizing healthcare needs, deploying culturally sensitive virtual care programs, and evaluating outcomes with **AI-powered analytics**. By leveraging AI in a systematic and inclusive manner, this approach addresses social determinants of health, improves equity, and enhances healthcare quality, offering a scalable model to improve health outcomes in geographically and demographically diverse settings.

The integration of artificial intelligence (AI) into healthcare delivery offers an innovative means to address the unique challenges of underserved and remote populations. Communities in northern Saskatchewan, Canada, where Indigenous populations face high rates of diabetes and other chronic conditions and shortages of healthcare providers, exemplify the pressing need for such advancements.^1^ AI technologies, particularly in virtual care platforms, hold promise for managing these conditions and bridging healthcare gaps. However, applying a uniform approach to these communities fails to consider the diverse demographic and clinical needs that shape healthcare requirements. A systematic, community-tailored framework is essential for creating effective, **AI-driven healthcare solutions** in these settings. Saskatchewan's rural and remote communities vary significantly in their demographic makeup and healthcare needs. Northern regions, with large Indigenous populations, face a disproportionately high burden of chronic diseases like tuberculosis, diabetes, and cardiovascular conditions, often accompanied by earlier onset and more severe complications.^2^ Meanwhile, other areas with higher concentrations of elderly or immigrant populations demand tailored approaches to geriatric care and culturally sensitive healthcare delivery.^3^ Virtual care platforms, when developed with an understanding of these demographic profiles, can address these disparities by ensuring relevance and accessibility to specific populations. Box 1.0 provides a **five-step approach** to integrate AI effectively into care delivery in remote and hard-to-reach populations. It offers a systematic method that aligns with core healthcare values of equity, accessibility, and quality, offering a transformative path to reducing disparities and improving the health of all populations.

Box 1.0. A five-step approach to integrate AI effectively into care delivery in remote and hard-to-reach populations

**1. Step 1: Build non-biased AI-enhanced community profiles**

Based on a two-eyed seeing approach,^4^ non-biased artificial intelligence (AI) algorithms analyze and compile comprehensive community profiles. Machine learning processes data on demographics, disease prevalence, healthcare infrastructure, and social determinants of health, identifying patterns and correlations that traditional methods might miss**.** For example, Health Catalyst's Population Health Analytics^5^ has been implemented to enhance care coordination and quality.^6^ AI-powered analytics have been used to identify care gaps and optimize resource allocation, demonstrating improved patient engagement and reduced emergency visits.^7^ These real-world applications suggest that such tools can be adapted to remote Canadian Indigenous communities to enhance healthcare access and equity.

**2. Step 2: Assess readiness for AI-integrated virtual care**

AI tools evaluate internet connectivity, digital literacy, and access to smart devices, forecasting potential barriers and recommending interventions like enhancing digital infrastructure and tailored digital literacy training. For example, secure tools like Education Perfect for Healthcare supports digital literacy.^8^ ​Bridging technological gaps in remote communities necessitates targeted infrastructure investments, such as broadband expansion, low-power AI models for offline use, and solar-powered health kiosks to address electricity and connectivity limitations. The Health Grid Sierra Leone project successfully provided solar power and internet connectivity to 26 health facilities in remote communities, enhancing healthcare access.^9^

**3. Step 3: Prioritize healthcare needs with AI decision support**

AI decision support systems prioritize communities with high disease rates and limited healthcare access by using weighted criteria to analyze factors such as health condition severity and local healthcare service availability, ensuring efficient resource allocation. AI-powered decision support systems can provide real-time suggestions to healthcare providers, aiding in clinical decision-making, with increased accuracy and reduced time and cost, thereby ensuring efficient resource allocation.^10^

**4. Step 4: Deploy AI-driven community programs**

Implementing AI-enhanced virtual care programs requires cultural sensitivity and alignment with community needs. AI-driven natural language processing (NLP) tools can develop culturally appropriate content and address language barriers, continuously adapting programs based on community feedback. AI-driven translation tools have been employed to create culturally tailored health education platforms, ensuring that healthcare information is accessible and relevant to diverse populations.^11^ These tools are designed for secure and compliant use in healthcare settings, ensuring that virtual care programs are both effective and culturally sensitive.​

**5. Step 5: Measure outcomes with AI analytics**

AI-driven predictive analytics tools track and measure healthcare program outcomes across clinical effectiveness, operational and economic efficiency, and patient satisfaction, allowing for continuous optimization of healthcare delivery. Key evaluation metrics include diagnostic accuracy, assessed through sensitivity and specificity to minimize misdiagnoses,^12^ and patient outcomes, measured by reduced readmission rates and improved recovery times.^13^ Operational efficiency, gauged through hospital throughput and length of stay, reflects AI's role in optimizing healthcare delivery,^14^ while the economic impact is assessed via cost savings and return on investment. A case study at Area 25 Health Center in Malawi demonstrated AI's potential, with fetal monitoring technology reducing stillbirths and neonatal deaths by 82% over three years.^15^ These metrics ensure that AI-powered healthcare solutions remain effective, efficient, and patient-centered. By leveraging these advanced analytics, healthcare organizations can refine programs over time, ensuring optimal performance and enhanced patient care.

A systematic approach begins with building **AI-driven comprehensive community profiles** that integrate real-time data on demographics, prevalent health conditions, and social determinants of health. Information such as income levels, educational attainment, and housing quality lays the foundation for identifying health disparities and planning targeted interventions.^16^ A culturally sensitive methodology based on a two-eye-seeing approach underpins this ideology and informs the course of action.^4^ This initial step is pivotal in ensuring that interventions address not just immediate healthcare needs but also the underlying factors affecting health outcomes. The next critical step is assessing digital readiness through **AI tools**, including evaluating internet connectivity, digital literacy, and access to technology. In many rural areas, inadequate digital infrastructure poses a significant barrier to adopting virtual healthcare.^17^ Addressing these gaps through initiatives such as **AI-driven digital literacy training**, improved internet infrastructure, and affordable access to devices ensures that virtual care systems are both accessible and equitable.^18^ Such efforts prevent technological advancements from exacerbating existing inequalities and pave the way for effective implementation.

**AI decision support systems** should be deployed to prioritize communities’ healthcare needs based on community profiles, ensuring that resources are allocated efficiently. However, implementing AI-driven healthcare in remote and Indigenous communities faces several challenges, such as (a) the high costs associated with AI technologies can be prohibitive for smaller healthcare facilities; therefore, establishing public–private partnerships and utilizing open-source AI frameworks can reduce expenses and facilitate adoption,^19^ (b) many rural and Indigenous communities lack digital infrastructure, including reliable internet, interoperable electronic health records, and AI-compatible medical equipment, which limits AI integration. Privacy and regulatory concerns in Indigenous healthcare require strong data sovereignty protections, ensuring community-led governance and ethical AI deployment. Investments in broadband expansion, secure AI systems, and culturally informed policies create the foundation for equitable and effective AI-driven healthcare in these settings; (c) the shortage of trained personnel and limited AI literacy among healthcare professionals can hinder implementation. Implementing interdisciplinary training programs and fostering collaboration among healthcare providers, data scientists, and IT professionals can enhance AI adoption and integration^20^ and (d) resistance from healthcare providers and community members can impede AI adoption in healthcare settings. Providers may fear job displacement and loss of clinical autonomy, while Indigenous communities may harbor mistrust due to historical injustices and concerns over data sovereignty. Addressing these issues requires transparent communication, co-designed AI models, and training programs that integrate Indigenous knowledge systems and align with community values. Addressing these barriers through targeted strategies is crucial for the successful deployment of AI-driven healthcare solutions in these communities.

However, despite its transformative potential, AI-driven healthcare is not without **limitations**, particularly in rural and Indigenous settings. A key concern is the risk of exacerbating existing health disparities by shifting emphasis from in-person care to digital services, inadvertently marginalizing populations that prefer or require in-person interactions with healthcare providers. AI models trained on non-representative datasets have been shown to perpetuate algorithmic biases, leading to inequitable healthcare predictions and resource allocation. AI, when trained on datasets that fail to account for the health needs of marginalized populations, including rural and Indigenous communities, can result in misdiagnoses and inappropriate treatment prioritization,^21^ hence failing to align with community-specific health priorities and gaps. Over-reliance on AI-driven solutions may contribute to workforce shortages by reallocating resources from human-driven care to digital platforms, reducing direct provider engagement, and increasing patient reliance on automated services that may not adequately support complex healthcare needs.^22^ This risk is particularly concerning for elders, individuals with low digital literacy, and patients with chronic conditions who require human oversight and culturally sensitive care.^23^ If improperly implemented, AI may further entrench healthcare inequities rather than resolve them. To mitigate these challenges, it is critical to adopt a hybrid model where AI serves as a complementary tool rather than a replacement for traditional healthcare services. Community-led AI development, with direct participation of Indigenous leaders, healthcare professionals, and local stakeholders, is essential to ensuring AI technologies align with the cultural, ethical, and contextual realities of the populations they aim to serve with deliberate efforts to avoid implicit bias.^24^ This is the approach we are following to provide virtual care services to remote Indigenous communities at the Virtual Health Hub in Saskatchewan.^25–27^

AI-driven healthcare delivery systems may use weighted criteria to evaluate the severity of health conditions, the availability of local healthcare services, and the potential impact of virtual care interventions. Communities with limited healthcare facilities and high rates of chronic diseases can benefit from remote monitoring and teleconsultation programs.^28^ This targeted allocation maximizes the impact of healthcare resources and prioritizes communities with the greatest need. Based on a two-eyed seeing approach,^4^ deploying **AI-driven community programs** to operate virtual care that is culturally sensitive and community-centric is essential for successful implementation.^4^ Engaging local stakeholders, including community leaders and healthcare providers, in the design and delivery of these programs ensures alignment with cultural practices and community-specific needs. For Indigenous communities, involving leadership in program development fosters trust and ensures that healthcare services respect cultural traditions and address unique challenges.^29^ Similarly, collaborating with immigrant populations can help identify and overcome language barriers and other cultural factors impacting healthcare access.^30^

Finally, the effectiveness of virtual care programs must be continuously evaluated to ensure sustainability and improvement. **AI-powered analytics** can track clinical, economic, and environmental metrics such as patient satisfaction, health outcomes, and cost-effectiveness. Ensuring the long-term sustainability of AI-driven healthcare requires continuous maintenance of digital infrastructure, ongoing training of healthcare providers, and iterative evolution of AI models. Infrastructure sustainability depends on regular system updates, cybersecurity enhancements, and broadband expansion efforts, particularly in remote regions where connectivity remains inconsistent. Equally important is ongoing AI training for healthcare professionals, ensuring that providers stay adept at interpreting AI outputs, troubleshooting issues, and integrating AI-assisted decision-making into patient care. AI systems must adapt over time to remain clinically relevant, and incorporating federated learning techniques can be beneficial. Federated learning enables AI models to evolve using decentralized, real-time patient data while preserving privacy and preventing bias drift. This approach allows multiple healthcare institutions to collaboratively train models without sharing sensitive patient data, thereby enhancing the adaptability and robustness of AI systems in clinical settings.^31^ Without continuous updates, static AI models risk becoming obsolete, limiting their effectiveness in dynamic healthcare environments. A sustainable AI ecosystem requires structured policy frameworks that support digital infrastructure upkeep, workforce training, and periodic AI model updates, as outlined in Canada's *Artificial Intelligence and Data Act (AIDA)*, which emphasizes regulatory measures to ensure responsible AI development and long-term sustainability.^32^

These insights allow for the refinement and optimization of programs over time. Moreover, documenting the reduction in carbon footprints from decreased travel for in-person visits underscores the broader environmental benefits of virtual healthcare systems. By following this structured approach, **AI-driven healthcare** can be tailored to the diverse needs of remote and underserved populations, ultimately improving health outcomes and ensuring equitable access to care. While this framework is tailored to Saskatchewan, its core principles—community co-development, data sovereignty, and AI transparency—can be adapted to other remote and Indigenous contexts across Canada and beyond. In Aotearoa, New Zealand, the application of Māori principles to AI design emphasizes cultural responsiveness and ethical governance, aligning with local values and priorities.^33^

AI deployment in Indigenous healthcare necessitates adherence to the CARE Principles (Collective Benefit, Authority to Control, Responsibility, Ethics), which emphasize Indigenous data sovereignty and community control over data usage.^34^ Implementing these principles ensures that AI systems align with community values and priorities. Additionally, addressing algorithmic bias is crucial, as biases in AI can disproportionately affect marginalized populations, leading to inaccurate predictions and exacerbating health disparities. Employing strategies to detect and mitigate such biases is essential for creating fair and equitable AI technologies in healthcare.^35^

## Conclusion

The deployment of AI-driven healthcare in remote and Indigenous communities presents a major opportunity to address long-standing inequities in access, diagnosis, treatment, and prevention. However, realizing this potential requires intentional and community-centered implementation that prioritizes equity, capacity building, infrastructure readiness, and cultural relevance. The proposed five-step framework highlights a structured, systematic and ethical approach to AI integration, emphasizing culturally sensitive development, digital inclusivity, and continuous evaluation. To prevent AI from becoming a technological divide rather than a bridge, sustained investments in broadband expansion, workforce training, and community-led AI design are essential. By embedding AI into healthcare systems with precision, oversight, and cultural responsiveness, this framework promotes AI enhancement rather than replacement of human-driven care, paving the way for sustainable and equitable healthcare transformation in Indigenous communities.
